# Cord blood banking: Balancing hype and hope in stem cell therapy

**DOI:** 10.18332/ejm/192930

**Published:** 2024-10-07

**Authors:** Agnieszka Bień, Joeri Vermeulen, Grażyna Bączek, Michalina Pięta, Beata Pięta

**Affiliations:** 1 Faculty of Health Sciences, Medical University of Lublin, Lublin, Poland; 2 Department of Life sciences and Medicine, Faculty of Science, Technology and Medicine, University of Luxembourg, Eschsur-Alzette, Grand Duchy of Luxembourg; 3 Department of Public Health, Biostatistics and Medical Informatics Research group, Vrije Universiteit Brussel, Brussels, Belgium; 4 Department of Obstetrics and Gynecology Didactics, Faculty of Health Sciences, Medical University of Warsaw, Warsaw, Poland; 5 University Clinical Hospital in Poznan, Poznan, Poland; 6 Department of Mother and Child Health, Poznan University of Medical Sciences, Poznan, Poland

**Keywords:** stem cells, cord blood banking, informed decision, pregnancy

In recent years, stem-cell therapy has become a promising and advanced research topic. The development of treatment methods has generated huge expectations. The wide range of possibilities for using stem cells renders this cutting-edge therapy a turning point in modern medicine and one that offers hope for incurable diseases^1^.

Umbilical cord blood is the biological material that remains in the umbilical cord and placenta after the birth and cutting of the umbilical cord. It is a valuable source of stem cells with high proliferative potential. Although in many countries until recently, it was treated as medical waste and disposed of together with the placenta and umbilical cord, it is now often collected to isolate stem cells for storage and later use. Umbilical cord blood is a rich source of hematopoietic stem cells, which, like bone marrow cells, can be transplanted to rebuild the hematopoietic and immune systems. They show greater efficacy than stem cells obtained from adult donors^2^. Stem cells are unspecialized cells in the human body, and one of their advantages is their ability to self-renew indefinitely, thus maintaining a constant cell population level in the body. These cells also can differentiate into specialized progenitor cell types. As a result of this process, they take on the morphological and biochemical properties that are necessary to perform specialized functions. The transition from an undifferentiated state to a fully differentiated state is gradual^1^.

In relation to their differentiation capacity, stem cells can be divided into the following groups: 1) totipotent cells, which originate from the blastocyst and have the capacity to differentiate into any cell type; 2) pluripotent cells, which can develop into any cell other than reproductive cells; and 3) unipotent including precursor cells with the potential to differentiate into only one cell type). With regard to origin, there are three types of stem cells, namely embryonic stem cells (totipotent or pluripotent), somatic stem cells found in the organs of adult organisms (pluripotent or unipotent), and stem cells from umbilical cord blood^2^. Following the first transplantation in 1988, cord blood has become the standard source of hematopoietic stem cells^3,4^. In 2006, the European Group for Bone Marrow Transplantation equated hematopoietic stem cells from umbilical cord blood with hematopoietic stem cells from bone marrow and peripheral blood after rHu-G-CSF mobilization; since that time, the indications for using hematopoietic stem cells for transplantation purposes have remained the same, regardless of their source^4^.

This editorial highlights the significance of umbilical cord blood banking and underscores the pivotal role of knowledge in decision-making. Women who are well-informed about umbilical cord blood banking are better equipped to make informed choices that align with their needs and those of their families. By emphasizing the importance of education and awareness, this editorial seeks to empower women to navigate the complexities of umbilical cord blood banking with confidence and clarity.

## The use of cord blood stem cells

Currently, cord blood stem cells are used to treat or alleviate about 80 diseases, particularly lymphatic and hematopoietic conditions such as sickle cell anemia, Fanconi anemia, and adrenoleukodystrophy. Ongoing clinical research continues to expand the therapeutic possibilities of these cells, exploring new disease entities and experimental therapies. This often improves the health of people struggling with neurodegenerative diseases, cancer, diabetes, immune deficiencies, thalassemia, and lupus erythematosus, among others. Cord blood is also used in experimental therapy for myocardial infarction or osteoporosis^2,5,6^.

Over the last years, cord blood use has expanded beyond transplant medicine into areas of regenerative medicine in clinical research trials for conditions once thought untreatable, such as autism, cerebral palsy, and brain injury^7,8^. There is also evidence that umbilical cord blood as a cell therapy, coupled with rehabilitation, is slightly more effective than rehabilitation alone for improving motor skills in children with cerebral palsy^9,10^.

Until recently, the main limitation regarding using cord blood stem cells was the limited number of hematopoietic cells per cord blood unit. In April 2023, the US Food and Drug Administration (FDA) approved a cord blood stem cell multiplication procedure. This nicotinamide-modified stem cell transplant derived from umbilical cord blood is being developed for treating cancers (for adults and children ≥12 years of age) of the hematopoietic system and hemoglobinopathies. Using this technology, the number of hematopoietic cells can be increased 50-fold^11,12^. The potential for clinical applications of human cord blood stem cells is leading to new research. As it progresses, the possibilities for its therapeutic use are also changing. These cells also represent a promising source for the production of gene therapy products, e.g. CAR-T (chimeric antigen receptor) and CAR-NK (chimeric antigen receptor natural killer) cells – ‘natural killer’ cells with an added receptor that enables them to destroy leukemic cells. However, current methods for producing these types of cells are very expensive^6^.

## Legal conditions of cord blood banking

The European Parliament has issued a resolution on voluntary and unpaid donation of tissues and cells, emphasizing ethical principles and policy recommendations to ensure the integrity, safety, and ethical conduct of the donation processes^13^. The resolution underscores the importance of non-remuneration, informed consent, and safeguarding health, advocating for transparent and safe donation systems with robust traceability and donor evaluation procedures. Policy recommendations include increasing public awareness, ensuring fair compensation for living donors, and promoting collaboration between public and private sector entities in tissue and cell banking. By adhering to these principles and policies, Member States can facilitate voluntary and unpaid donations while protecting the rights and well-being of donors and recipients.

Article 29 of the European regulation underscores the necessity of informing parents about the various options related to cord blood donation at birth, encompassing public or private storage, donation for autologous or heterologous purposes, or research, and mandates the provision of objective and accurate information regarding the advantages and disadvantages of cord blood banks. Simultaneously, Article 30 emphasizes the importance of enhancing parental rights to informed consent and freedom of choice concerning cord blood stem cell preservation practices. Furthermore, Article 33 calls for an update to the opinion issued by the European Group of Ethics in Science and New Technologies in 2004 on the ‘Ethical aspects of umbilical cord blood banking’ (Opinion No. 19) in light of advancements in cord blood stem cell preservation and ongoing clinical trials. The ethical framework proposed by the group includes principles such as respect for human dignity and integrity, autonomy, justice, solidarity, beneficence, non-maleficence, and proportionality, aiming to ensure ethical considerations guide decisions in this field^14^.

The European Group on Ethics’ opinion on commercial cord blood banking is critical of the practice. While it does not advocate for its prohibition, the opinion raises concerns about the ethical implications, such as the commodification of the human body and potential inequities in access to future therapies^14^. It also emphasizes the importance of solidarity and social equity in healthcare, suggesting support for public cord blood banks to ensure fair access and consumer protection. Despite acknowledging the tension between freedom of enterprise and the need for regulation, the opinion leans towards discouraging commercial banking practices while advocating for informed consent and strict oversight^15^.

Most discussion has, to date, focused on two topics: informed consent for collection, banking, and use; and the debate between those who favor public storage for altruistic purposes and those who advocate private storage for autologous use. There is generally agreement or consensus in the guidelines that donations for public storage should be supported and encouraged in hospitals where possible. Given the consensus in national and international guidance on these two issues, it is time to examine other ethical issues in greater detail. These include additional uses of cord blood units, for example, for research or the production of blood-derived drugs, and the economic implications arising from the extensive international network for exchanging cord blood for transplantation^16^.

## Cross-border case studies

Although European Union Directives provide a Union-wide framework of regulations, individual member countries have adopted varying approaches in national legislation to regulate cord blood banking^17^. The regulatory landscape surrounding cord blood banking varies across European countries. For example, in several countries, the procedure is governed by specific laws.

In Belgium, cord blood storage is permitted only for philanthropic allogeneic or directed use, governed by specific laws prohibiting the storage and use of cord blood for purposes not supported by scientific evidence or therapeutic goals. Similarly, France has regulations limiting cord blood preservation for proven therapeutic needs or altruistic purposes, with bans on commercial blood banks and strict requisites for authorized facilities. However, some countries like Germany allow both non-profit public and private banks, albeit with guidelines emphasizing limited indications for autologous preservation. In the United Kingdom, cord blood collection is regulated by the Human Tissue Authority, with several non-profit banks for allogeneic use and innovative mixed public-private banking models introduced to balance potential therapeutic benefits with ethical considerations. While European countries share common objectives in regulating cord blood banking, the specific approaches and allowances vary, reflecting diverse ethical and scientific considerations^17^.

In Italy, umbilical cord blood banking is organized through public hospital-based facilities, coordinated by the Italian Cord Blood Network, established in 2009. This network promotes allogeneic unrelated voluntary cord blood donation and manages cord blood collection, storage, and distribution for clinical indications. Currently, 18 facilities comply with national regulations and international standards, aiming to achieve an optimal inventory of 60000 units to ensure broad access to hematopoietic transplantation. Despite challenges such as cost maintenance and declining clinical use, Italy continues to invest in research and regulation to develop new cord blood components^18^.

In Spain, six public cord blood banks operate under the guidance of the Spanish Bone Marrow Donor Registry. A national guide aims to standardize collection, banking, and release methods, with recent objectives focusing on meeting inventory targets, implementing quality criteria, and promoting research on new therapeutic uses of cord blood. Despite a substantial inventory, utilization rates remain relatively low, prompting efforts to develop new therapeutic products from units not suitable for transplantation. Regulatory clarification has been provided for certain cord blood products, such as autologous plasma derivatives. Still, challenges persist in harmonizing regulations, particularly regarding classifying cord blood components as blood products or medicinal products, which affects their regulatory oversight and approval processes^18^.

## Cord blood banking regulations

In the early years of developing cord blood banks, 1990s and early 2000s, cord blood banking was not properly regulated. Therefore, no barriers were set in place to launch such an activity. In the field of family banks, it led to the rapid development of providers of that activity, which were not running real banks but were focusing on commercial activity. At the same time, all procedures related to cell and tissue procurement were outsourced to other banks. As an effect of that, in some markets, there were dozens of active companies promoting cord blood banking services. Sometimes, this led to misleading marketing, and even more importantly, there was a lack of proper control over the samples collected and sent cross-border. Over time, implementing more strict regulations and the financial crisis caused banks to consolidate. Nowadays, cord blood banking is fully regulated, and parents have access to trustworthy institutions. It is worth mentioning that despite EU directives in place, each EU member state regulates cord blood banking on its own. The New EU Regulation for Substance of Human Origin of the European Union will provide more harmonization in the field. It is currently under preparation, and it is expected to come into force within a couple of years.

**Table 1:** Summaries and comparisons of different aspects of regulations* about cord blood banking in six selected countries

*Germany*	*Spain*	*Switzerland*	*Hungary*	*Poland*	*Italy*
Banks must be accredited by local authorities for respective Federal State and the central authority. Since cord blood is treated as a pharmaceutical product, requirements toward banks are more strict than in other countries – similar to pharmaceutical manufacturing. Every hospital where cord blood is collected, needs to be separately accredited by authorities, and needs to have a contract with the accredited bank. Hospital personnel need to be regularly trained on how to collect cord blood. Samples can be collected only for accredited banks.	Banks who offer cord blood banking services need to be authorized with each collection center (hospital) according to the Royal Decree-Law. It establishes quality and safety standards for the donation, obtaining, evaluation, processing, preservation, storage and distribution of human cells. In addition, each cord blood bank has to be authorized by each health ministry of each region in the country. Samples stored in Spain have to be available for unrelated too. Most banks are offering storage abroad.	Every entity which deals with cells and tissues, needs to fulfil the requirements of the Transplantation Act. Requirements for banks differ on the basis of purpose of banking (autologous vs allogeneic). It is allowed for accredited entities to send collected samples cross-border after obtaining permission.	Cord blood banking is fully regulated by local law. Banks need to follow the same rules as other entities involved in transplantations of cells and organs. Samples can be send abroad under certain conditions, and that can be done by an entity which is licensed by local authorities.	Activity of banks are regulated by the Transplantation Law. Banks need to be accredited, inspected and follow regular audits. Only banks accredited by Polish authorities can offer services. The bank is responsible for collection sites, it is obliged to have a contract between bank, hospital and collecting person. Regular trainings and reporting are mandatory. Exporting collecting samples is possible for accredited banks.	Family banking is possible only in situations when there is a patient with set diagnosis that collected cord blood can be used for him. If there is no such case, it is allowed to organize individual collection in the hospital using a collection kit provided by one of the companies and then send it to a bank abroad. Permission needs to be organized by parents.

* Once banks are offering additional cryopreservation of other perinatal tissues such as umbilical cord, placenta, and amniotic membranes, other regulations may apply.

## Providing information on blood banking

There is a scarcity of studies delving into healthcare professionals’ knowledge and communication practices concerning cord blood banking for expectant parents. Future investigations should prioritize exploring healthcare professionals’ knowledge, attitudes, and practices in this realm, alongside scrutinizing how such knowledge shapes their professional conduct during childbirth. Understanding these dynamics is crucial as they can affect the information relayed to expectant parents, either enhancing or hindering their decision-making process^19^. Conversely, a study exploring pregnant women’s awareness of cord blood stem cells, and their attitude regarding banking options in France, Germany, Italy, Spain, and the United Kingdom revealed a strong preference for public banking in all five countries based on converging values such as solidarity. Attitudes of pregnant women are not an obstacle to the rapid expansion of allogeneic banking in these European countries. Banking choices do not appear to be correlated with household income. The extent of commercial marketing of cord blood banks in mass media highlights the importance of obstetricians and midwives playing a central role in raising women’s awareness early during their pregnancy with evidence-based medical information about banking options^20^. However, healthcare professionals should assume that pregnant women often lack sufficient information about cord blood banking. The decision-making process should be conducted to ensure every pregnant woman has the opportunity to make a well-informed decision about cord blood banking^21^.

Information on the possibility of cord blood banking and cord stem cell banking is an important element of antenatal education, as reflected in the European Parliament Resolution of 11 September 2012 (2011/2193(INI). Acknowledging the role of cord blood stem cells, the European Parliament called on the Member States of the European Union to provide better protection for the rights of parents to make informed decisions and exercise freedom of choice regarding the possibility of securing cord blood during childbirth^13^.

While making decisions about cord blood banking, consumers must be presented with up-to-date, evidence-based information about the likelihood of private banking resulting in benefits. Nevertheless, donating cord blood to a private or public bank should be an autonomous decision of parents. Disclosures should also consider the opportunity costs of storing privately instead of donating to a public bank and possibly contributing to a life-saving therapy for someone else^22^. Informed choice and signed consent are crucial elements in cord blood donation, whether to a public or private bank. Given that consent should ideally be obtained before childbirth, informing women about cord blood banking during prenatal care is imperative, a task well-suited for obstetricians, midwives, and family physicians. Providing accurate information about the potential benefits and concerns of cord blood banking allows women to make informed decisions. Educational opportunities for healthcare providers and patients are essential to facilitate this process. A checklist could aid pregnant women in selecting their preferences regarding cord blood banking options, including acceptable uses of donated cord blood, contact preferences, and involvement in future programs. Fostering transparency and accountability requires open discussions between women and their healthcare providers regarding cord blood banking^23^. Private cord blood bank marketing that advertises hypothetical future treatments can be misleading and may influence consumer behavior. This marketing may breach existing advertising laws. Regulatory bodies should enforce the law to help prevent public health and personal financial harm^22^.

## Collecting umbilical cord blood

Cord blood collection is an aseptic procedure that is performed after vaginal childbirth or cesarean section. International guidelines on the timing of umbilical cord blood collection after birth include recommendations on timing the umbilical cord blood collection to obtain the optimal amount of blood. However, in line with current standards, the medical staff always make the decision on when to cut the umbilical cord and should not be influenced by the fact of cord blood collection^24^.

According to the recommendations of the American College of Obstetricians and Gynecologists, delaying the moment of clamping the umbilical cord following the birth of term newborns, increases hemoglobin levels and boosts iron stores in the first few months of life, which may have a beneficial effect on the child’s subsequent development. Benefits are also observed in premature babies, as delayed cord clamping leads to improved circulation, increased red blood cell volume, reduced need for blood transfusion, and reduced incidence of necrotizing enterocolitis and intraventricular hemorrhage in this group of babies. Given these benefits, in agreement with other professional organizations, the American College of Obstetricians and Gynecologists recommends delaying umbilical cord clamping in healthy newborns and preterm infants by 30–60 seconds after birth. However, the Society does express caution that the moment of cutting the cord should not be delayed beyond the recommended time standards, as there has been a slight increase in the incidence of jaundice that subsequently requires phototherapy in term neonates who have had delayed umbilical cord clamping. Therefore, it is important to ensure that mechanisms are in place to monitor and treat neonatal jaundice in a given facility. Studies have also indicated that delayed umbilical cord clamping does not increase the risk of postpartum hemorrhage^25^.

The American Academy of Pediatrics has also endorsed these recommendations. However, it was emphasized that due to the diversity of practices, the team should decide to care for the mother-child dyad^24^. The decision to cut the umbilical cord should take into account clinical situations in which immediate cord clamping should be considered, or care should be individualized, e.g. hemorrhage, hemodynamic instability, abnormal placentation, e.g. previa, abruption, placental circulation not intact, e.g. abruption, previa, cord avulsion, intrauterine growth restriction with an abnormal cord Doppler evaluation^25^.

According to the International Federation of Gynecology and Obstetrics guidelines, delayed clamping of the umbilical cord in the first minute in preterm infants born before 34 weeks gestation improves neonatal hematological parameters and may reduce mortality without increasing the risk of subsequent complications. It appears to improve short- and long-term outcomes in children born at term and four years of age, which shows beneficial outcomes in the fine motor and social domains. However, there is insufficient evidence to indicate which delay duration is best. Current evidence confirms that for preterm births, the umbilical cord should not be clamped before 30 seconds. Future studies could compare different lengths of delay. Until then, 30 seconds to 3 minutes is considered reasonable, or until the umbilical cord becomes limp and pale^26^. Preterm and term-born infants should be allowed to breathe freely during the period in which umbilical cord clamping is delayed. Additional research is needed to assess the long-term effects on the child related to the timing of umbilical cord clamping and brain development^24,27^.

According to WHO recommendations (2014), the umbilical cord should not be clamped earlier than the first minute after birth for term or preterm infants to improve maternal and neonatal outcomes^24,28^.

The American College of Nurse-Midwives has stated that delayed clamping of the umbilical cord (once the umbilical artery pulsations have ceased) should be the standard of care in all birthing facilities for term and preterm births^29^. Consequently, the Royal College of Obstetricians and Gynaecologists (RCOG) recommends deferred umbilical cord cutting for healthy newborns and preterm babies until at least 2 minutes after birth. The term ‘deferred’ is preferred, as it suggests a planned policy instead of delayed, which may mean later than ideal^30^. In contrast, the National Institute of Clinical Excellence (NICE) guidelines indicate that the umbilical cord should not be clamped earlier than 1 minute after birth unless there are concerns about the integrity of the umbilical cord or the baby’s heart rate is less than 60 beats/min and not accelerating^31^.

## Cord blood banking

Once collected, stem cells are stored by a specialist cord blood bank, which can be private, public, or hybrid.

Private – these banks offer cord blood storage services to be used exclusively by the child’s family. The parents cover all costs associated with collecting, preparing, and storing the cells. The blood is stored for private use by the family in question, who can use it in the future should the child or other family members need stem cell-based treatment. Parents can also use such blood for regenerative medicine in clinical trials or medical treatment experiments. If blood from the family bank is used, the transplant center obtains the stem cells for the recipient free of charge^32^.

Public – these banks collect, process, and store cord blood indefinitely for anyone who needs it for medical purposes. The blood donor remains anonymous. Units available in the public bank are cataloged, and the records can be searched nationally and internationally. Handling fees are charged to cover part of the storage and administration costs. Public banks receive other funding through philanthropic donations, government funds, and grants. Public cord blood banks are run according to altruistic motives and are usually non-profit entities. If a family donates their child’s cord blood to a public bank, the donation may save a life. However, there is no guarantee that they will recover the blood later for their own use because public banks focus on storing the largest units, so 85–90% of collected samples are not stored^33^.

Hybrid – combines features of both public and private cord blood banks with different solutions to reduce costs for the private payer. These banks offer the option of donating part of the cord blood to the public pool and storing some of the blood for their own use. This option gives parents some flexibility, enabling them to support the community through public cord blood banking while retaining the ability to use the stored blood for their own needs^32,34^.

The choice of cord blood bank depends on parental preference (altruistic vs motivation to protect own family orientation), availability (in most hospitals, public donation is not possible), budget, and an assessment of the risks and benefits of storing cord blood for oneself or other family members^20^. It is also recommended to consult a physician or medical professional for more information and advice on choosing the right cord blood bank. Each type should be controlled by the local Ministry of Health or an entity under its authority^32^. Cord blood banks in the United States, whether private or public, are periodically inspected for compliance with standards. Similar regulations also exist in the European Union. In 2019, the board of directors of the Cord Blood Association adopted the first ever ‘Model Criteria for the Regulation of Cord Blood Banks and Cord Blood Banking’. These guidelines set out detailed criteria for quality management, informed donor consent, pre-selection and testing, collection, processing, shipping and transport, quality measurement outcomes, and data sharing^11^.

Donating cord blood to a family or public bank is an autonomous decision parents make. The role of a midwife is to inform them about the possibility of securing cord blood after birth and the current therapeutic applications of cord blood stem cells based on up-to-date, evidence-based knowledge. With access to reliable information, parents can make an informed decision about whether to have cord blood collected after birth. Another important role of the midwife is to conduct a preliminary interview to assess donor eligibility. After birth, the midwife is responsible for the appropriate collection of cord blood, packing the kit, and ensuring it is swiftly delivered to the laboratory to initiate testing and preparation procedures.

## Conclusion

Correct collection, transport, testing, and preparation of cord blood play an important role in its proper deposition and use in treating many diseases. An important aspect of the use of stem cells is the creation of unambiguous regulations governing the status of stem cells while guaranteeing safety and monitoring each stage of their use. Numerous studies additionally pointed out the need to inform both parents-to-be and healthcare professionals about the possibility of collecting, depositing, and using stem cells derived from cord blood. It is vitally important for parents to be able to make informed choices. For this to happen, the information provided must be accurate, objective, up-to-date, and evidence-based^35^. Providing evidence-based information contributes to parent-centered care and helps parents make informed decisions on the option that best suits their family’s situation, values, and concerns^36^. Midwives, in turn, should be well-versed in the current guidelines and recommendations for both cord clamping time and cord blood collection procedures, ensuring these practices are consistently implemented in their daily care.

## Data Availability

Data sharing is not applicable to this article as no new data were created.
